# Drug-Induced Systemic Lupus Erythematosus: A Rare Presentation of Hydralazine-Induced Lupus

**DOI:** 10.7759/cureus.72069

**Published:** 2024-10-21

**Authors:** Renard Jerome, Johnny S Randhawa, Sondos Badran, Sylvia Li, Niki Mohammadi

**Affiliations:** 1 Internal Medicine, Arrowhead Regional Medical Center, Colton, USA

**Keywords:** drug-induced lupus, drug-induced lupus (dil), hydralazine-induced lupus syndrome, rare side effect, rheumatology

## Abstract

Systemic lupus erythematosus (SLE) is a chronic autoimmune disorder characterized by the production of autoantibodies directed against nuclear and cytoplasmic antigens. SLE can be induced by various medications, such as hydralazine, procainamide, isoniazid, methyldopa, chlorpromazine, quinidine, and minocycline. Hydralazine-induced lupus syndrome was first reported in 1953, and only occurs in 5-10% of patients taking hydralazine. We present a case of a 76-year-old female with a past medical history of chronic kidney disease (CKD) stage II, hyperlipidemia, primary hypertension, and type II diabetes mellitus who was initially admitted for complaints of pre-syncope. Initial chest X-ray demonstrated right lower lobe lung opacity with small pleural effusion, and computed tomography (CT) chest with intravenous (IV) contrast was negative for pulmonary emboli but showed small bilateral effusions and a small pericardial effusion. A transthoracic echo (TTE) was performed and demonstrated an ejection fraction of 65% and no signs of pericardial effusion. CBC was remarkable for pancytopenia with a notable drop in all cell lines compared to her baseline from prior admissions. Antinuclear antibody (ANA) titer elevated at 1:1280, and anti-histone titers were positive. Medication reconciliation was performed, and hydralazine was discontinued with marked improvement in her clinical course. The patient had a bone marrow biopsy performed, and the results were negative for any myeloproliferative or myelodysplastic changes. The patient was then discharged with outpatient follow-up with hematology/oncology and rheumatology.

## Introduction

Systemic lupus erythematosus (SLE) can be induced by various medications, such as hydralazine, procainamide, isoniazid, methyldopa, chlorpromazine, quinidine, and minocycline [1]. Hydralazine-induced lupus syndrome was first reported in 1953 and only occurs in 5-10% of patients taking hydralazine [2]. Drug-induced lupus (DIL) is an autoimmune phenomenon where drug exposure leads to the development of SLE such as clinical features [3].

The most common symptoms of DIL include fever, arthralgias/arthritis, myalgias, rash, and/or serositis. More severe manifestations, such as kidney disease and central nervous system involvement, are uncommon but can still be present. DIL usually develops only after months and years of exposure to the offending agent, although latencies of days or weeks have been described in some instances [4].

## Case presentation

Our patient is a 76-year-old female with a history of CKD stage II, hypertension, hyperlipidemia, and type II diabetes mellitus who presented with sensations of passing out after stepping out of the shower. The patient reported two similar episodes in the past and endorsed fever, chills, fatigue, and temperature insensitivity for the past six months. The patient also added unintentional weight loss of approximately 11.34 kg secondary to poor oral intake and dysphagia. The patient denied any chest pain, shortness of breath, nausea, vomiting, headache, or changes in vision or hearing prior to the episode of pre-syncope.

**Figure 1:**
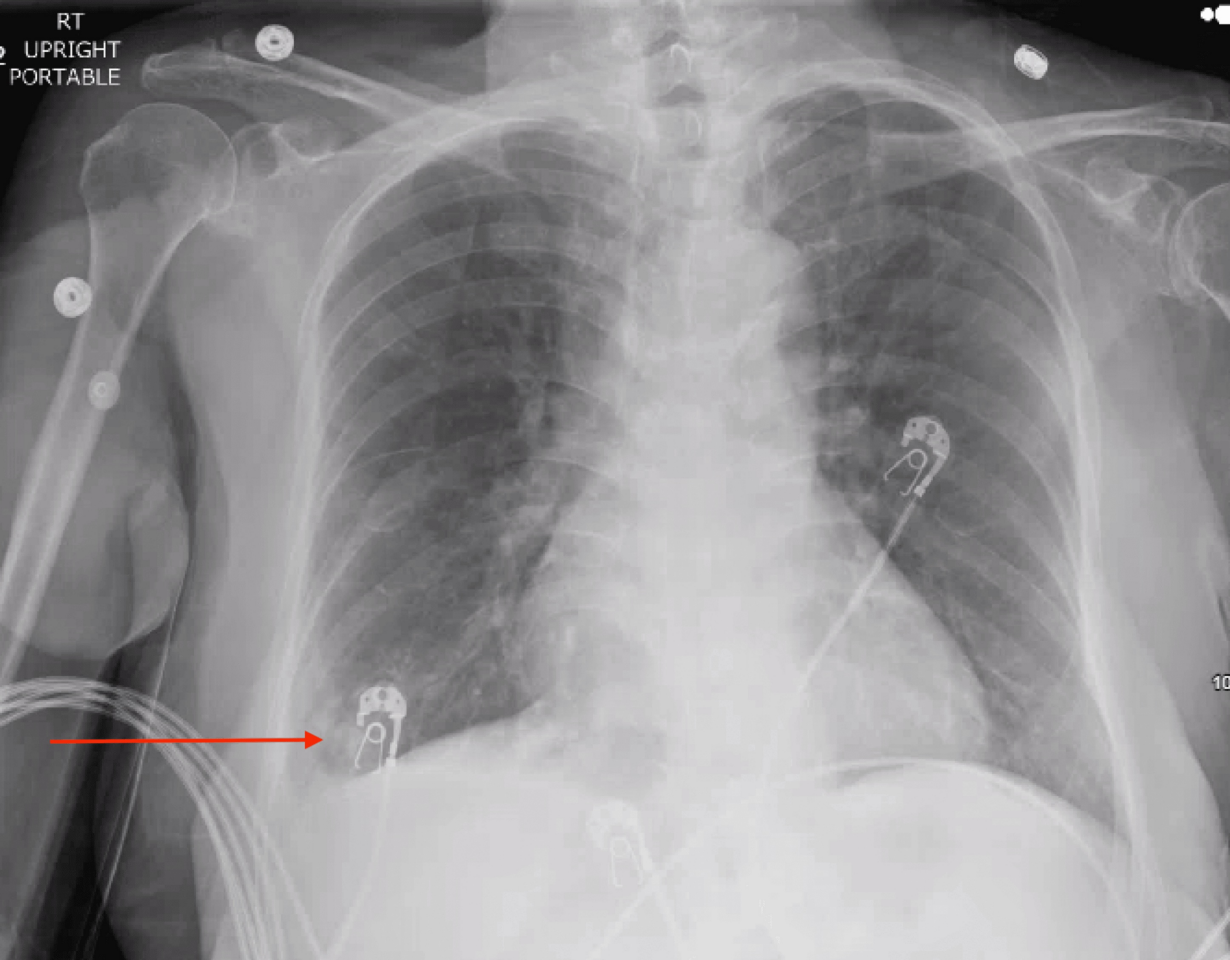
Anterior-posterior chest radiograph showing right lower lobe lung opacity with small pleural effusion

**Figure 2:**
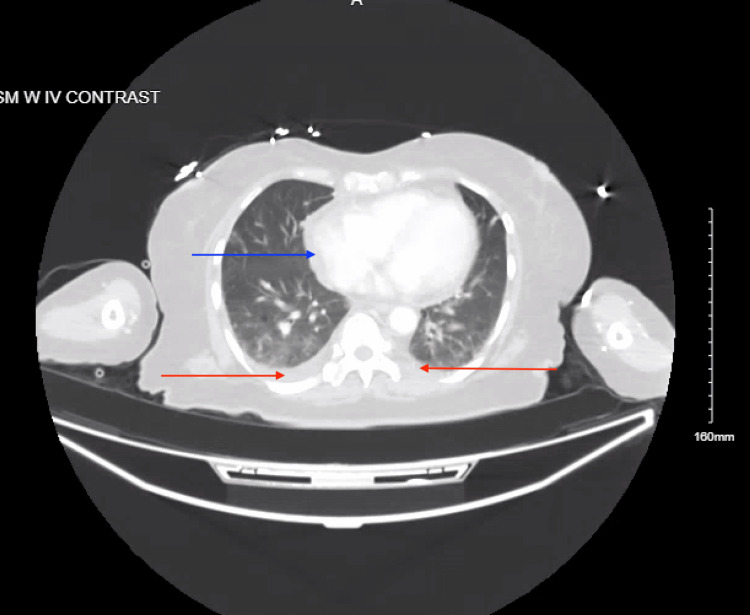
Axial section of a chest computed tomography with IV contrast demonstrating small bilateral pleural effusions (red arrows) and small pericardial effusion (blue arrow)

In the emergency department, the patient’s blood pressure was 155/72 mmHg, heart rate was 115 beats per minute, respiratory rate was 16 breaths per minute, and temperature of 37.1 °C (98.7 °F), saturating 96% on room air. The physical exam was remarkable for brisk pulses, otherwise within normal limits. Comprehensive metabolic panel (CMP) was significant for mild hyponatremia of 131 mmol/L, anion gap metabolic acidosis with serum bicarbonate of 15 milliequivalents per liter (mEq/L) and an anion gap of 18, elevated AST 65 U/L and an acute kidney injury on chronic kidney disease (AKI on CKD) with eGFR of 45.2 mL/min/1.73 m^2^, and serum creatinine of 1.24 milligrams per deciliter (mg/dL) with baseline of 0.72 mg/dL one year prior. Complete blood count (CBC) showed white blood cell of 1.1 10^3^/uL and normocytic anemia with hemoglobin of 7.5 grams per deciliter (g/dL) and mean corpuscular volume (MCV) of 82 fL, with previously noted baseline hemoglobin of 12.3 g/dL approximately one year ago. The fecal occult blood test (FOBT) was negative, and troponin T was also elevated at 29.8 ng/L. C-reactive protein (CRP) was elevated at 3.67 mg/dL with a d-dimer over 10,000 g/mL d-dimer units (DDU). Thyroid-stimulating hormone (TSH) was within normal limits at 2.12 mIU/L. Biofire respiratory panel was negative, and serum lactate was within reference ranges. Initial chest X-ray showed right lower lobe lung opacity with small pleural effusion (Figure 1). Computed tomography (CT) head without IV contrast showed no acute intracranial abnormalities, and CT chest with IV contrast demonstrated bilateral small pleural effusions and a small pericardial effusion, but no pulmonary embolism (Figure 2).

CT abdomen and pelvis with IV contrast showed no acute abnormalities. Anti-double stranded DNA antibody was positive, with titers of 1:40. C3 complements were low at 45 mg/dL, and C4 was normal at 18 mg/dL. Kappa and lambda free light chain ratio was within normal limits of 1.18, and the lupus anticoagulant antibody screening test was positive. Hematology/oncology service was consulted, who recommended a bone marrow biopsy to rule out any primary bone marrow disorders and additional serum testing (i.e., serum vitamin B12/folate/ferritin levels). Serum B12, folate, and ferritin were within normal limits. Anti-histone antibody was noted to be strongly positive at 4 HU. Flow cytometry demonstrated 73% granulocytic cells, 16% lymphocytoid cells, and 4% monocytoid cells and was unremarkable for monoclonality or phenotypic abnormalities. A bone marrow biopsy was performed and demonstrated a variably normocellular marrow with 20-30% cellularity and active trilineage hematopoiesis. The cytomorphology showed reactive changes and megakaryocytes with small hypolobated forms with slight nuclear irregularity and atypia, raising the possibility of association with a drug reaction, autoimmune conditions such as SLE or immune thrombocytopenia (ITP), or kidney disease. A thorough medication reconciliation was performed and the patient's home antihypertensive Hydralazine 100 milligrams twice daily was held due to concerns for drug-induced lupus. The patient was noted to have slow incremental improvements in her cell lines immediately one day after admission and was subsequently discharged home with outpatient follow-up with her primary care physician. The patient was also recommended to follow up with hematology/oncology and rheumatology and avoid any future exposures to hydralazine.

## Discussion

Hydralazine-induced lupus (HIL) is well-researched and uncommon; it affects a small proportion of patients receiving hydralazine medication. HIL occurs in less than 10% of patients taking hydralazine, its incidence increasing in a dose-dependent manner [5,6]. This ailment might be difficult to diagnose because of its rarity, as well as its inconsistent presentation and latency duration [6].

A constellation of symptoms, including pre-syncope, fever, chills, exhaustion, and a notable unintended weight loss, was observed in our patient. These non-specific symptoms were originally puzzling, especially when combined with pancytopenia and serositis. Our focus shifted to autoimmune etiologies after the bone marrow biopsy's negative myeloproliferative and myelodysplastic results ruled out primary hematologic malignancies. This example illustrates the diagnostic quagmire frequently seen in drug-induced lupus, wherein initial presentations may resemble more prevalent disorders such as infections, cancers, or primary autoimmune diseases [7].

An increased ANA titer and a positive anti-histone antibody were essential for diagnosing DIL. Although not totally specific, anti-histone antibodies are very sensitive to DIL and have been shown to be present in idiopathic SLE as well. The diagnosis was further supported by the patient's improvement after stopping hydralazine, which the patient had been taking since June 2022. This case emphasizes how important it is to correlate the results of thorough antibody testing with clinical history and drug use in individuals with suspected DIL [8,9].

As our patient's case illustrates, the main strategy for managing DIL is to stop using the offending substance. After quitting hydralazine, her clinical symptoms and hematologic abnormalities significantly improved. To check for lingering or recurrent symptoms and to make sure that any possible autoimmune or hematologic disorders are not missed, long-term follow-up is crucial. Comprehensive care requires coordination across rheumatology, hematology, and primary care [10].

## Conclusions

We emphasized the uncommon presentation of HIL in this case report, underscoring the need to consider drug-induced systemic lupus erythematosus as a possible diagnosis in patients exhibiting non-specific symptoms. Significant clinical improvement resulted from stopping the offending medicine, hydralazine, highlighting the necessity of a comprehensive medication evaluation in situations like these. To guarantee a total cure and to monitor for any recurrence of symptoms, long-term follow-up is essential via a multidisciplinary approach, including follow-up between rheumatology, hematology, and primary care.
